# Authentication of Meat and Meat Products Using Triacylglycerols Profiling and by DNA Analysis

**DOI:** 10.3390/foods9091269

**Published:** 2020-09-10

**Authors:** Vojtech Hrbek, Kamila Zdenkova, Diliara Jilkova, Eliska Cermakova, Monika Jiru, Katerina Demnerova, Jana Pulkrabova, Jana Hajslova

**Affiliations:** 1 Department of Food Analysis and Nutrition, University of Chemistry and Technology, Technicka 5, 166 28 Prague 6, Czech Republic; Vojtech.Hrbek@vscht.cz (V.H.); Monika.Jiru@vscht.cz (M.J.); Jana.Pulkrabova@vscht.cz (J.P.); Jana.Hajslova@vscht.cz (J.H.); 2 Department of Biochemistry and Microbiology, University of Chemistry and Technology, Technicka 5, 166 28 Prague 6, Czech Republic; Eliska2.Cermakova@vscht.cz (E.C.); Katerina.Demnerova@vscht.cz (K.D.); 3 Research Department, Food Research Institute Prague, Radiová 1285/7, 10231 Prague 10, Czech Republic; Diliara.Akhatova@vupp.cz

**Keywords:** meat, authentication, triacylglycerols, ambient mass spectrometry, DNA, PCR

## Abstract

Two alternative, complementary analytical strategies were successfully used to identify the most common meat species—beef, pork and chicken—in meat products. The first innovative high-throughput approach was based on triacylglycerols fingerprinting by direct analysis in real time coupled with high-resolution mass spectrometry (DART–HRMS). The second was the classic commonly used DNA analysis based on the use of nuclear or mitochondrial DNA in multiplex polymerase chain reaction (mPCR). The DART–HRMS method represents a rapid, high throughput screening method and was shown to have a good potential for the authentication of meat products. Nevertheless, it should be noted that due to a limited number of samples in this pilot study, we present here a proof of concept. More samples must be analyzed by DART–HRMS to build a robust classification model applicable for reliable authentication. To verify the DART–HRMS results, all samples were analyzed by PCRs. Good compliance in samples classification was documented. In routine practice under these conditions, screening based on DART–HRMS could be used for identification of suspect samples, which could be then examined and validated by accurate PCRs. In this way, saving of both labor and cost could be achieved. In the final phase, commercially available meat products from the Czech market were tested using this new strategy. Canned meats—typical Czech sausages and luncheon meats, all with declared content of beef, pork and chicken meat—were used. Compliance with the label declaration was confirmed and no adulteration was found.

## 1. Introduction

The adulteration of food is a current socioeconomic worldwide problem. Consumer demand for correct and understandable food labeling is growing. The informed choice of the products that they want to buy is issue of high concern.

One relatively common fraudulent practice is the replacement or dilution of a highly valuable commodity by a cheaper one. This problem may be encountered in meat products—specifically in minced ones. Pork, beef or chicken meat are among the most popular and nutritionally valuable food commodities. Nevertheless, as chicken meat is the cheapest, it may be used to substitute for expensive beef. In addition to economic fraud, hazard for consumers suffering of allergic reaction to certain meats (e.g., chicken protein allergy) must be considered. Moreover, religious aspects may be of concern, as eating pork is not acceptable in Muslim populations.

With regards to the above facts, analytical strategies that can quickly, affordably and reliably detect such unfair practices are urgently needed. A number of approaches have been employed for authentication purposes. The most common of them is target analysis of specific markers such as nucleic acids, peptides/proteins or metabolites. Wide variety of techniques including chromatographic, electrophoretic, spectroscopic immunochemical and molecular–biologic methods have been used for meat authentication [1,2,3,4,5]. In routine practice also immunological methods based on the interaction between an antigen and antibody (mainly ELISA) are used for authentication of animal species in meat products.

Various molecular techniques used for food authentication have been developed and reviewed [6,7]. These techniques are based on the DNA polymorphism between species and are classified into three types. (1) polymerase chain reaction (PCR)-based techniques, (2) hybridization-based techniques and (3) sequencing-based techniques such as DNA barcoding to analyze short standard DNA sequences and forensically informative nucleotide sequencing FINS [5,8,9,10,11,12,13,14]. Various PCR methods have been described: endpoint PCR, real-time qPCR and digital dPCR that allow the amplification of a chosen region of genomic or mitochondrial DNA. In animal cells, mitochondrial DNA (mtDNA) is present in many copies, while genomic DNA is mostly present in one copy. Genomic DNA is an appropriate target for quantification, while a small addition of undeclared species to the sample can be demonstrated by the detection of mtDNA [12].

Regarding specific markers screening by chromatographic methods, in our recent study, we demonstrated the possibility to recognize addition of 2% chicken meet addition to pork by assessing specific ratios of amino acids 1-methylhisitidine/3-methylhistidine [15]. However, this approach is rather time-consuming. The same applies e.g., for proteomics-based procedures in which meat species authenticity is performed by means of well-defined proteogenomic annotation, carefully chosen surrogate tryptic peptides ad analysis using a high resolution mass spectrometry HRMS [16]. This technique, thanks to high spectral resolution, enables skipping over chromatographic separation. The use of ambient ionization method such as direct analysis in real time (DART) allows a great simplification and increase in the speed of mass spectrometry-based measurements. In particular case, the sample investigation can be performed in the open environment of the laboratory by its introduction into the ionization region, where it is exposed to a stream of ionizing medium [17]. The attractive features of DART have made this technique, apart of other applications, a challenging tool rapid characterization of food composition and/or assessment of its authenticity based on metabolomic fingerprinting [18]. For processing of data generated by DART–HRMS advanced statistical methods represented by principal component analysis (PCA) followed by discriminant analysis, e.g., partial least squares discriminant analysis (PLS-DA) are commonly used.

The objective of this pilot study was to demonstrate the applicability of a new authentication strategy for large sets of selected meat products enabling both labor and cost saving. In the first phase, samples with suspect triacylglycerols (TAGs) profile were rapidly identified by DART–HRMS technique, in the next step, the confirmation was performed by validated PCRs. To our knowledge, this is the first study that analyzes meat samples by both mentioned methods and discusses their discrimination potential.

## 2. Materials and Methods

### 2.1. Samples

Samples were purchased through the retail network of the Czech Republic. In total, 36 samples were available: pork (*n* = 3), chicken (*n* = 3), beef (*n* = 3) and heat-treated meat products: ham (*n* = 3), sausages (*n* = 11), luncheon meat (*n* = 7) and meat in its natural juices (*n* = 6). Approximately 200-g of each sample was homogenized by means of an electric grinder (Grindomix GM 200, Retsch, Düsseldorf, Germany) and stored in a plastic container at −20 °C until analysis. The fat content and meat composition of the commercial meat products are shown in Table 1. All samples were tested using both analytical approaches, DART–HRMS–represents a rapid screening method and PCR–verification method. DART–HRMS models for data evaluation were created from nine samples of meat (pork, chicken and beef), 27 samples (meat products) were used for comparison of use in practice. It must be mentioned, that due to the very low number of analyzed samples, this innovative strategy was being tested to determine the potential of this method for the purpose of authentication of meat and meat products. The purpose of this pilot study was to present the suitability of this combination; it is proof of the concept.

### 2.2. Direct Analysis in Real Time Coupled with High-Resolution Mass Spectrometry (DART–HRMS) Analysis

#### 2.2.1. Sample Preparation for Instrumental Analysis

6 mL of hexane was added to the homogenized sample (2 g) in a 15-mL plastic tube. The sample in the plastic tube was extracted for 1 min using a Turrax instrument (T 10 basic ULTRA-TURRAX^®^, IKA, Staufen, Germany). After centrifugation (5 min, 20 °C, 10,000 rpm), the extract was transferred to a glass vial and ready for DART–HRMS analysis. (QC) 100 μL of each pure meat hexane extract (beef, chicken and pork) was mixed as a quality control sample.

#### 2.2.2. Conditions of DART–HRMS Analysis

For the analysis using ambient mass spectrometry, the DART ion source (DART-SVP) was fitted with a 12Dip-It^TM^ tip scanner autosampler (IonSense, Saugus, MA, USA) coupled to an Exactive^TM^ benchtop (Thermo Fisher Scientific, Bremen, Germany). A Vapur^TM^ interface (IonSense, Saugus, MA, USA) was employed to couple the ion source to the mass spectrometer and low vacuum in the interface chamber was maintained with a membrane pump (Vacuubrand, Wertheim, Germany). The distance between the exit of the DART gun and the ceramic transfer tube of the Vapur was set to 10 mm, the gap between the ceramic tube and the inlet to the heated capillary of the Exactive was 2 mm.

The DART and MS instruments were operated in positive ionization mode and the optimized settings were as follows: helium pressure: 5.5 bar; gas temperature: 450 °C; discharge needle voltage: 1000 V; grid electrode: 250 V. For mass spectrometric detection, the settings were as follows: capillary voltage: 60 V; tube lens voltage: 120 V; capillary temperature: 250 °C. The sheath, auxiliary, and sweep gases were disabled during DART–MS analysis.

The mass spectrometer was operated at a mass resolving power of 50,000 FWHM calculated for *m*/*z* 200. The mass spectra acquisition rate was 2 spectra s^−1^. Liquid samples were delivered into the DART ionization region with the use of a 12 Dip-It tip scanner autosampler. Dip-It^TM^ tips (IonSense, Saugus, MA, USA) were inserted into a holder. μL of hexane extract of each sample were individually placed on the end of glass tips. The Dip-It holder was mounted on the body of the autosampler and the Dip-It tips were automatically moved at a constant speed of 0.5 mms^−1^ through the helium gas between the exit of the DART gun and the inlet of the Vapur interface.

Standard external mass calibration of the MS system in the range of 50–1000 *m*/*z* was performed in positive mode prior to the measurement of every sample set (sequence) according to the manufacturer’s instructions. Moreover, an adjusted mass calibration for ESI(+) in the mass range of *m*/*z* 50–1000 using collision-induced dissociation (CID) at 25 eV was subsequently performed to cover the lower masses.

#### 2.2.3. Data Analysis

Chemometric analysis included multivariate data analysis using unsupervised and supervised models. Principal component analysis (PCA) and partial least squares discriminant analysis (PLS-DA) were employed based on SIMCA software (v. 13.0, 2011, Umetrics, Umea, Sweden; www.umetrics.com).

In the first stage, data processing and data pretreatment must be carried out to capture the bulk of the variation between different datasets. In this way, raw data generated by meat samples analysis employing the DART–HRMS technique (TAGs signals in positive ionization mode) in the form of absolute peak intensities were preprocessed using a constant row sum, that is, each variable was divided in the sum of all variables for each sample; this procedure transformed all the data to a uniform range of variability. In other words, the intensities of the variables obtained from the profile of the analyzed sample were summed and then each specific variable was divided by the sum thus obtained, thus avoiding the different intensities of the individual ions which would be caused by the measurement itself and not by the differences of analyzed samples. DART–HRMS data were initially processed with the software Xcalibur 2.2 and copied to MS Excel 2010. The macro function was used in the following step to create the final tables which were exported to the SIMCA software.

Subsequently, Pareto scaling was applied prior to PCA and PLS-DA [19]. Then, PCA analysis enabled the transformation of the original variables (normalized intensities of ions) to the new uncorrelated variables (principal components). In this way, the reduced dimensionality of the data were obtained while still preserving information from the original data set. Additionally, PLS-DA was subsequently applied to identify and reveal the most significant TAGs. PLS-DA was performed to provide a better distribution of samples and enable the creation of a statistical model and validation.

The quality of the models was evaluated by the goodness-of-hit parameter (R^2^X), the proportion of the variance of the response variable that is explained by the model (R^2^Y) and the predictive ability parameter (Q^2^), which was calculated by a k-fold internal cross-validation of the data using a default option of the SIMCA software. In general terms, the value of R^2^ must be higher than Q^2^ and an acceptable value of Q^2^ is more than 0.5 [20]. In addition, the models were also evaluated in terms of their recognition and prediction abilities. Recognition ability represents the percentage of samples in the training set that were correctly classified. Prediction ability is the percentage of samples in the test set that are correctly classified by using the model developed during the training step. For this purpose, seven-fold internal cross-validation was used [21]. For the control of the Q^2^ values, if they were stable and relevant (correctly calculated), the permutation test was used [22].

S-plot illustrating the distribution of the detected features involved in the statistical evaluation was used as a tool for ´marker´ selection. Features at the extremes of the S-plot, the outermost ions can be considered as ‘markers’ with the highest importance for sample separation. For sorting the ‘markers’ according their importance, a VIP (variable importance in projection) plot that explains X and correlates to Y can be used. The most important variables in a given model are those with VIP score >1. The other tool for explaining/confirming ions as markers is a variable line plot, which illustrates the variability among the top ions across the sample sets.

The tentative identification of compounds behind the marker ions was based on the estimation/calculation of the elemental formula (accurate mass and mass error for respective *m*/*z* values in MS^1^ and isotopic pattern were considered). The estimated molecular structure of the markers was compared with online databases such as ChemSpider (www.chemspider.com) or Metlin (www.metlin.scripps.edu/index.php).

#### 2.2.4. Quality Control

To verify the absence of carryover effects and to control the stability of the recorded fingerprints, blank and quality control (QC) matrix samples were analyzed within the DART–HRMS sequence. It should be noted that the order of the tested samples within the sequence was random (established based on random number generation) to avoid any possible time-dependent changes during DART–HRMS analysis, which could result in false clustering. To check the overall performance of the instrumental system, QC samples were inserted into the sequence, always after a set of ten tested samples and analyzed under the same conditions. The QC sample was a pool of all meat sample extracts. In this way, the repeatability of sample fingerprints could be monitored. The good instrument performance was documented by a tight clustering of these QC samples (i.e., the similarity of their fingerprints) in the PCA plot.

### 2.3. Analysis by Polymerase Chain Reaction (PCR)

Multiplex mPCRs were used for the authentication of meat origin. The design of this study was as follows: after homogenization of the meat or whole meat product, the isolation of the DNA was performed, followed by PCRs. The mPCR based on mitochondrial cytochrome b gene amplification was used for qualitative analysis. Two mqPCRs (triplex and duplex) were used, based on the amplification of a single copy of chromosomally encoded gene sequences. Single-copy chromosomal genes were analyzed, such as cyclic phosphodiesterase for cattle, beta actin for pigs; interleukin-2 (Il-2) for chickens and the myostatin gene for mammals and poultry.

#### 2.3.1. DNA Isolation

DNA was isolated from 200-mg homogenized samples (Section 2.1) using a cetyltrimethylammonium bromide (CTAB) method [23]. The quality of the isolated DNA was verified by 1% horizontal agarose electrophoresis in Tris/Borate/EDTA buffer (Bio-Rad, Hercules, CA, USA), DNA concentration and purity was determined spectrophotometrically with a nanophotometer (Implen, Munich, Germany).

#### 2.3.2. Primers and Probes

The primers and probes used are shown in Table 2 and were synthetized by East Port (Prague, Czech Republic).

#### 2.3.3. Multiplex mPCR

For this method, primers complementary to mitochondrial DNA cytochrome b were used [12,24]. mPCR amplification was conducted in 15 µL 1.5-mM MgCl_2_, 0.2-mM dNTP mix (Promega, Madison, WI, USA), primer mix (Metabion International AG, Planegg, Germany), 100 ng template DNA and 0.4 U Platinum^TM^ DNA polymerase (Thermo Fisher Scientific, Waltham, MA, USA). The primers were mixed in the ratio of 1:0.6:0.6:1.5:1.5 for SIM:B:P:C:H and used together to mPCR (ratio 1 means concentration 0.4 μmol∙L^−1^). Amplifications were performed in a Biometra T-Gradient (Whatman Biometra, Göttingen, Germany) as follows: initial denaturation at 94 °C for 2 min, 40 cycles of denaturation at 94 °C for 30 s, annealing at 53 °C for 30 s and extension at 72 °C for 30 s, final polymerization was for 5 min at 72 °C. Visualization and detection of amplicons were done on 2.5% agarose gel.

#### 2.3.4. Multiplex mqPCRs

Primers and probes used for mqPCR were complementary to single-copy chromosomally encoded gene sequences. The reaction conditions for pork and beef were adopted from Iwobi et al. [11], chicken mqPCR from Zdenkova et al. [24]. The analyses were performed in an ABI 7500 (Applied Biosystems™, Foster City, CA, USA), the 7500 Software v2.0.6 was employed for data analysis. Four fluorescence channels were analyzed separately.

The result of the triplex qPCR was amplification of the 104-bp-long bovine gDNA segment with the Texas Red fluorescence curve; 107 bp from pork gDNA with a fluorescence curve of the HEX fluorophore and a 97-bp-long amplicon from the gDNA of mammals and poultry with the FAM fluorophore. The duplex qPCR amplified the 135-bp-long amplicon of chicken gDNA with the TAMRA fluorophore together with a 97-bp-long amplicon from the gDNA of mammals and poultry with the FAM fluorophore.

#### 2.3.5. Data Analysis

To separate the PCR amplicons, a 2.5% agarose gel was used. The confirmation of amplicon size was based on comparing the length of the amplicons obtained from the samples with the length of the marker fragments (100-bp DNA ladder, New England Biolabs, Ipswich, MA, USA) and the positive control during the reaction (target DNA) which, together with the non-template control, was included in each amplification reaction.

The qPCR data analyses are based on evaluating the fluorescence curves of the amplification cycle. If the fluorescence value of the sample exceeds the base fluorescence value, the amplification is positively evaluated, and the sample thus contains the target segment. If the sample does not contain a target section or its sample content is lower than the detection limit of the method used, the fluorescence reading does not exceed the fluorescence baseline.

#### 2.3.6. Quality Control

PCR controls were performed for each reaction; a positive control containing the target DNA and no-template control without any DNA added.

## 3. Results and Discussion

In this study, TAGs and DNA were used for identification of the meat origin. The workflow was as follows: In the first step, the potential of using metabolomics profiling, focusing on the analysis of TAGs, employing DART–HRMS to differentiate pork, beef and chicken samples was investigated. The aim was to design the conditions of the analysis, which would allow obtaining separate groups for different meats on the PCA and PLS-DA plots. If such conditions would not have been found, the authentication of meat cannot be done with this approach. As the samples were differentiated according to the type of meat, the same strategy for the analysis of commercial meat products was used. At the same time, the results obtained by the DART–HRMS method were confirmed by the established validated mPCRs. A theoretical comparison of the two methods used for our analyses was also performed. A possible combination of these methods that can facilitate the routine analysis of meat and meat products was suggested based on both theoretical and practical comparisons. See the chapters below for more details.

### 3.1. Results of DART–HRMS Analysis

Hexane was chosen as an extracting agent for sample preparation, since it is a nonpolar solvent suitable for the isolation of TAGs. Other solvents (e.g., methanol) were tested. However, when compiling the statistical models, the assumption was confirmed that, for pure meat, methanol and hexane can be used for the extraction and analysis with excellent classification of different types of meats. However, the classification is no longer satisfactory with methanol when analyzing meat products containing other ingredients (besides meat, e.g., spices). The reason for this is the co-isolation of many other substances that come from other ingredients used in the production of meat products and may be different for different products. Therefore, the only possible way to use the metabolomic approach for the authentication of meat in a meat product is TAGs analysis. The profiles of TAGs associated with various types of meat should be the same in the original meat as in the final meat product, as shown below.

#### 3.1.1. DART–HRMS Fingerprints of Different Meat Types

**Figure 1:**
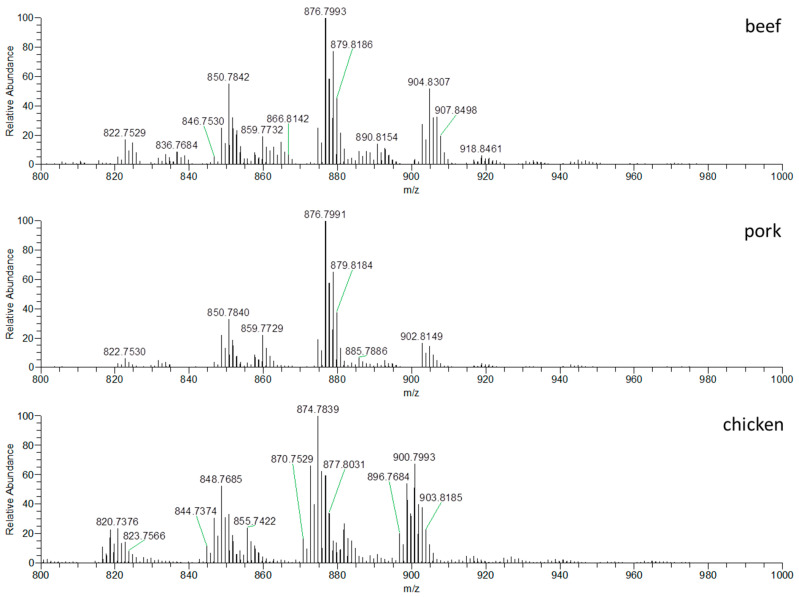
DART–HRMS profiles of triacylglycerols in hexane extract of beef, pork and chicken meat, *m*/*z* range 800–1000, positive ionization.

Figure 1 shows the characteristic fingerprints, TAGs profiles, associated with all three types of analyzed meat (beef, pork and chicken). TAGs form [*M* + NH_4_]^+^ ions, which are in the *m*/*z* range 800–1000. Apparent differences in TAGs profiles of individual meat species are evident, especially in the ratios of relative intensities of the individual TAG ions present in the profiles. Different TAGs are dominant in various types of meat, for example, TAGs with *m*/*z* 848.7682, 874.7837 and 900.7996 are predominantly found in chicken meat, while TAGs with ions with 850.7836, 876.7995 and 902.8148 are predominantly found in pork and ions with *m*/*z* 850.7836, 876.7995 and 904.8306 in beef. The mentioned differences are mainly in terms of the ratios of ions relative intensities, as shown in Figure 1. This fact is particularly important from the point of view to reveal the economically motivated adulteration, i.e., to reveal the addition of undeclared cheap chicken meat, more often to pork meat products, but also to beef products. Due to the differences in the TAGs present and their ratios, in particular meat profiles, the employed DART–HRMS strategy indicates a good potential for detecting adulteration.

**Figure 2:**
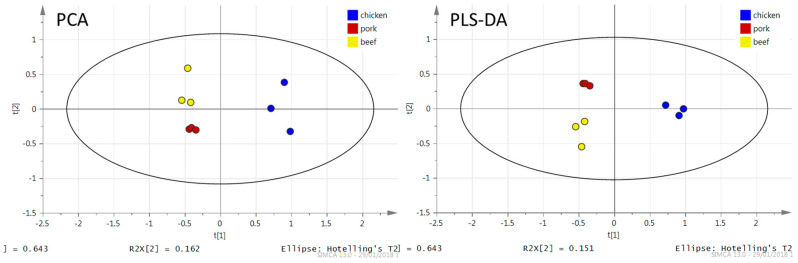
Principal component analysis (PCA) and partial least squares discriminant analysis (PLS-DA) plot created from ions related to triacylglycerols present in hexane extracts of chicken meat (blue), pork (red) and beef (yellow).

The fingerprint was converted to an ion list according to *m*/*z* and information about the intensities of the ions. The total number of detected ions related to TAGs in the profiles with signal intensity higher than 1000 cps was 40. However, it means 40 different summary formulas, but thanks to the isomers, the number of TAGs is probably higher. Data were transferred to MS Excel and TAG ions with VIP values higher than 1 (see Section 2.2.3) with their intensities were selected using the MAKRO function. The total number of selected ions was 15 (see Table 3). Furthermore, SIMCA v13.0 software was used for chemometric analysis. First of all, PCA was performed, followed by partial least squares discriminant analysis (PLS-DA). For the statistical analysis, 15 selected ions corresponding to TAGs were used. The obtained PCA and PLS-DA plots are shown in Figure 2.

As for the processing of DART–HRMS data, PCA clearly separated the meat varieties based on their TAGs profiles. In Figure 2 presenting the positive ionization data, PC1 and PC2 together described 79% of the sample set variability (64% and 15% for PC1 and PC2, respectively). Considering the fact that the first five PCs explain 99% (ESI+) of the total variance, The PC1/PC2 plot seemed to be a good starting point for sample clustering according to meat variety.

In the next step (following PCA analysis), PLS-DA was used, see Figure 2. As expected, efficient separation of samples into groups was achieved, and the mathematical model (R^2^X = 0.994, R^2^Y = 0.989, Q^2^ = 0.973) obtained in this way reliably enabled the correct classification of an unknown sample; recognition ability (100%) and prediction ability (100%) were excellent.

Although the model was created from a small number of samples, the differences between the samples of each species were noticeable. There is a certain risk of the model overfitting when using a small number of samples, for these reasons the model was control using a permutation test. It ought to be mentioned, that a larger number of samples would make the model probably more precise. In case of DART–HRMS the accuracy is secured by exact mass measurement and low mass differences (Δppm). Furthermore, the specificity is based on exact mass measurement and selection of appropriate *m*/*z* values. Estimation of sensitivity was based on the lowest intensity of selected ions (1000 cps). This corresponds to addition of approximately 3% of different meat into the other meat species. However, in the case of this study, the DART–HRMS method was designed and tested for screening purposes. The obtained results suggested that our DART–HRMS protocol could be used for rapid testing of a large number of samples. For precise authentication of suspicious or unclear samples, the use of validated PCR method is recommended.

#### 3.1.2. DART–HRMS Analysis of Real Meat Products

**Figure 3:**
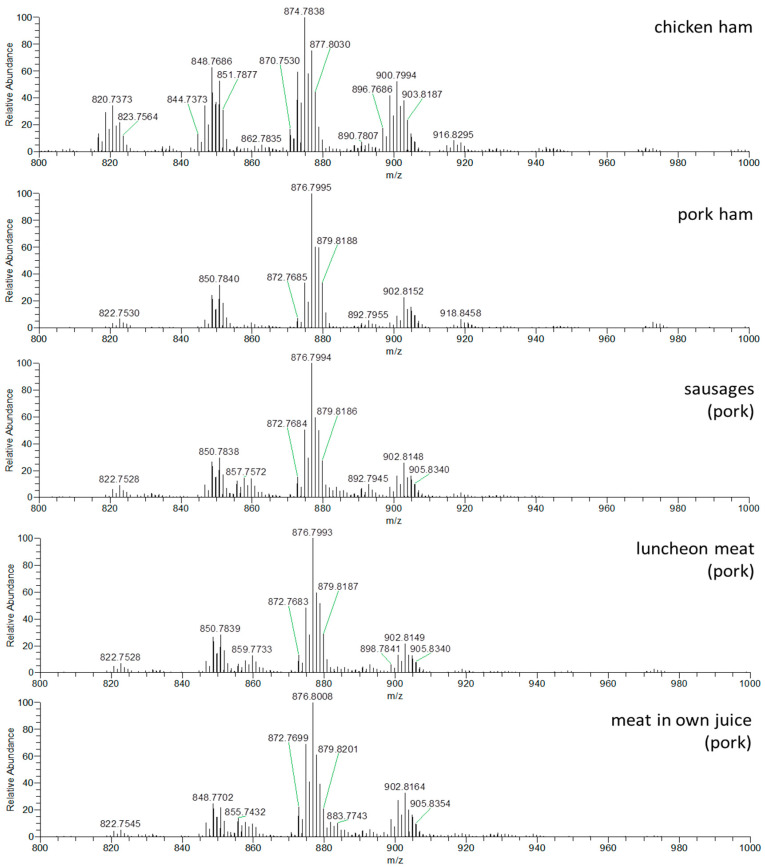
DART–HRMS profiles of triacylglycerol and hexane extracts of chicken ham, pork ham, sausages, luncheon meat and meat in natural juices, *m*/*z* range 800–1000, positive ionization.

The next step was application of suggested process for real meat products control. As representative meat products, sausages (as a traditional meat product consumed in the Czech Republic and other countries) and luncheon meat (as a product with high meat content), were selected for the verification of the declared meat type on packing. In addition, samples of pork and chicken ham-meat products containing almost no other ingredients (only pure meat) were analyzed as references. From all of these products, the hexane extracts, including lipophilic fraction with TAGs, were prepared and to obtain the TAGs profiles DART–HRMS was used, Figure 3.

The sample preparation chosen for the purposes of this study was affected as little as possible by the composition (ingredients) of the analyzed meat products. The DART–HRMS method was based on the analysis of TAGs, which are isolated from samples by a very nonpolar solvent (hexane). Such a sample preparation procedure naturally discriminates against the influence of inorganic salts in particular, which are rather polar in nature. Nonpolar substances could be a problem, but as this is not a quantification of individual substances, this is not a significant problem. Even if there is a possible suppression of the signal due to the lack of separation, all monitored TAGs would be suppressed similarly. Due to the use of data normalization during data processing, this effect should be compensated.

**Figure 4:**
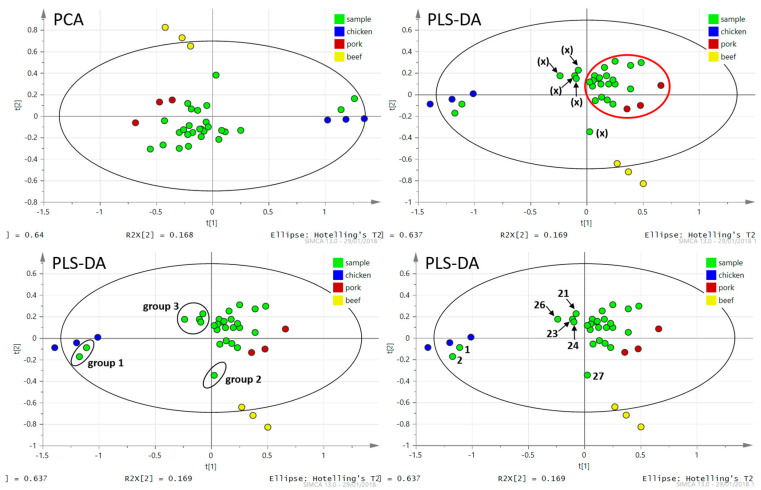
PCA and PLS-DA plots created from ions related to triacylglycerols present in hexane extracts of meat products (green), chicken (blue), pork (red) and beef (yellow). Group 1—chicken ham samples; group 2—meat products containing 70% of beef; group 3—pork meat products containing mechanically separated chicken meat and the rest of the green points represent meat products based on pork meat.

The PCA and PLS-DA chemometric analyses for data processing were used in the same way as it was mentioned in previous Section 3.1.1 (see Figure 4). As input data, the same TAGs (*n* = 15, see Table 3) were used, as in the case of chemometric analysis of data related to pure meat samples extracts. The PCA analysis focused on PC1 and PC2, which accounted for 81% of the sample set variability (64% and 17% for PC1 and PC2, respectively). Since the first five PCs explain 94% of the total variance, The PC1/PC2 plot seemed to be again a good starting point for sample classification.

The PLS-DA plot shows the classification of all three types of meat and meat products together. Most meat products samples were very close to the group of samples corresponding to pork meat. The values of the coefficients of recognition and prediction had a low value (R^2^X = 0.806, R^2^Y = 0.434, Q^2^ = 0.329), because the plot includes four groups of samples. However, the classification of meat samples into groups by type of meat was perfect, and the attribution of meat product samples to pork meat samples matched the declaration on the packaging. Two samples (chicken ham), marked with the number 1 (Figure 4), were separated from the rest of the meat products. They were very close to chicken meat samples, which again reflected the declaration on the packaging. The sample marked with number 2 (meat in natural juices, sample number 27)—near the group of beef samples (Figure 4)—contained 70% beef, which again corresponds with the meat declaration on the packaging.

The strategy presented in this article was based on the use of the rapid screening method DART–HRMS, which after data processing and visualization using a PCA or PLS-DA model will show suspicious samples in terms of authentication. Sample number 2 was an example of a “suspicious sample”. The sample was detected from among a group of authentic samples (reference samples) and thus became suspicious. It was necessary to verify the authenticity using the exact PCR method. In our case, the content of pork and beef was indicated on the packaging of the product. The position of the sample in the model corresponded to its composition, and thus the functionality of the model was verified. For an unknown sample, said PCR analysis could be additionally performed.

It can be very difficult to identify a sample as suspicious based only on a visual assessment. Therefore, the PCR method was used to confirm the results of DART–HRMS analyses. All samples were analyzed by PCRs. Thanks to these analyses, it was possible to determine the boundary (bold red line) in the PLS-DA model behind which suspicious samples are located, and it is necessary to subject these samples to confirmatory PCR analysis. Other samples that are located from the border (bold red line) towards the reference samples are considered as authentic samples containing only pork, as it demonstrated in Figure 4.

It is worth noting that four pork meat products (marked (x) that contained mechanically separated chicken meat tended to separate from the meat product made only from pork meat. They were situated towards the chicken meat samples in the PCA and PLS-DA plot, marked with the number 3 (see Figure 4). The other analyzed meat products mainly contained pork (without beef or chicken meat addition) were assigned to a group of authentic pork meat samples. From the analyses carried out and the obtained PLS-DA model, it is clear that this procedure correctly evaluates and assigns samples into groups according to the meat composition, the type of meat used in the production of meat products (sausages, luncheon meat, meat in natural juices). TAGs profiling by DART–HRMS could be used as a screening method to verify the composition of meat in meat products and to detect of adulteration by chicken meat.

**Figure 5:**
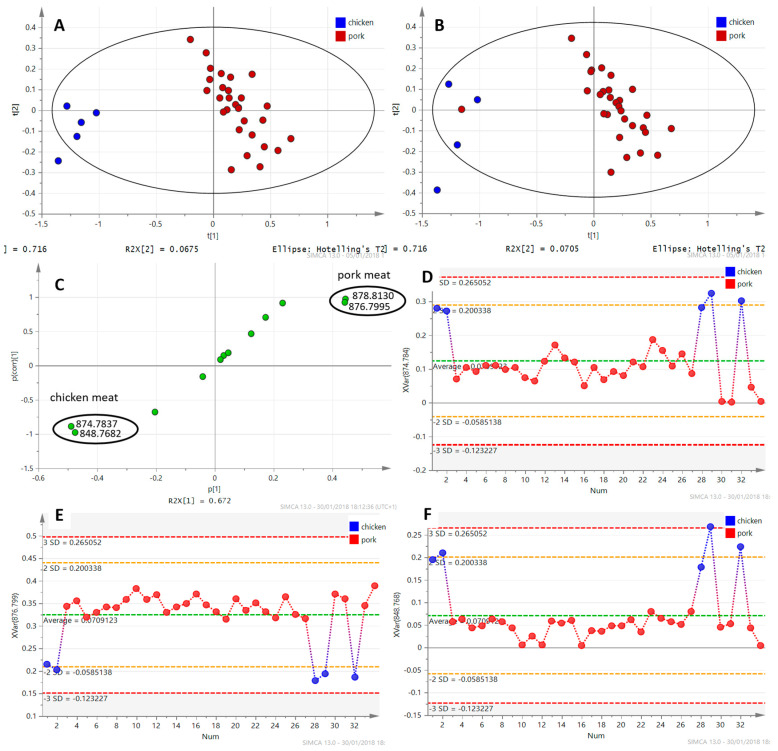
Examples of chemometric analysis of DART–HRMS data obtained from measurement of hexane extract of chicken and pork samples (meat and meat products). (**A**) PLS-DA plot; (**B**) PLS-DA plot illustrating the situation where the sample is adulterated; (**C**) S-plot and important chicken and pork markers; (**D**) variable line plot (trend plot) for ion *m*/*z* 874.7837—chicken meat marker; (**E**) variable line plot ion *m*/*z* 876.7995—pork meat marker; (**F**) variable line plot ion *m*/*z* 848.7682–chicken meat marker. Group of samples marked by number 1—four pork meat products which contained mechanically separated chicken meat.

To better demonstrate/simulate pork adulteration by chicken meat (the situation is similar for beef adulterated by chicken meat), PLS-DA plot (Figure 5A) was created using only the data for samples of chicken and pork and meat products composed of them. The plots show an excellent separation of chicken and pork samples (R^2^X = 0.784, R^2^Y = 0.929, Q^2^ = 0.894, recognition ability = 100% and prediction ability = 100%). In case of adulteration, in the sense of mislabeling, i.e., a meat product labeled as a pork product contains (undeclared) chicken meat, the sample would be assigned more to the chicken meat group. For better understanding, one of the chicken samples was marked as a pork sample. The model did not place it among the other pork samples (red dots), but correctly assigned/kept it in the chicken meat samples group (red dot among the blue dots group—chicken meat samples), as shown in Figure 5B.

To visualize ions that can be considered as ´markers´ with the highest importance for sample separation, several plots were created from the acquired data. Figure 5 shows an example of an S-plot (Figure 5C) created from the data obtained from pork and chicken meat (TAGs ions, [*M* + NH_4_]^+^), which were analyzed by DART–HRMS in positive ionization mode. Four the most remote ions visualized in the S-plot (highlighted with black circles), which also had the highest values in the VIP-plot, were selected as ´markers´. In fingerprinting-based authentication strategies, the identification of the detected metabolome components is not essential for sample separation. On the other hand, under some conditions, e.g., when only marker ions are considered for sample profiling, then the identification of unique markers may be of interest. The identification was based on comparing their estimated elemental composition, mass difference (Δppm) and isotopic profiles with the data available in online libraries and scientific papers [28,29]. The variable line plot for the ions at *m*/*z* 874.7837, 848.7682 (markers for chicken meat, Figure 5D,F) and *m*/*z* 876.7995 (marker for pork meat, Figure 5E) illustrates the changes in the content of the respective TAGs in the pork and chicken meat. The estimated elemental composition of these ions together with their tentative identification are shown in Table 3, where the markers also for beef are included. These ions are not markers in the true sense of the word, because they are not unique to one group (meat type). However, due to the fact that statistical data processing takes into account not only the presence and intensity of the ions, but also their relative ratio, these selected ions are essential for authentication. For beef, pork and chicken, significant (very abundant) ions were selected for each type of meat, which are determinants and key for verifying an undeclared addition of—for example—chicken meat into a meat product or determining which type of meat was used.

It could be concluded that the usage of supervised PLS-DA model could lead to a distortion of the separation (overfitting). This model was used for completeness and, also for better visualization of the obtained data and explanation of the context. The PLS-DA model is very similar as the PCA model in terms of sample separation. Permutation tests were also performed during data processing and confirmed its applicability. Due to the fact, that DART–HRMS analysis is intended primarily for rapid screening of large number of samples and identification of only suspicious samples for an additional accurate PCR method, it is possible to use the PCA model for this purpose.

#### 3.1.3. Confirmation of Isolated DNA Quality and Quantity

DNA was isolated using a CTAB method according to ČSN EN ISO 21,571 (ISO 21571, 2005). Both the yield and quality of the DNA obtained from pure musculature of beef, pork, chicken, turkey and horse were higher than 90 ng∙μL^−1^ for all tested, commercially important meat species. More than 100 ng∙μL^−1^ DNA was obtained from chicken, pork and beef samples. More than 50 ng∙μL^−1^ DNA was isolated from 26 real meat products; the highest yield (316 ng∙μL^−1^) was obtained from meat in its natural juices (sample number 22). The lowest yield of DNA was isolated from one sausage (sample number 6), 15 ng∙μL^−1^ was isolated with the ratio of absorbance (A 260 nm/A 280 nm) equal to 1.6 corresponding to nucleic acids with the presence of proteins. DNA of appropriate quality as well as quantity for subsequent amplifications was isolated from all samples tested.

#### 3.1.4. DNA Analysis of Meat and Meat Products

**Figure 6:**
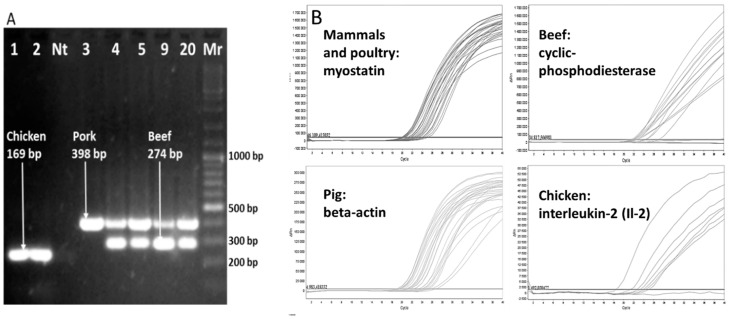
Examples of DNA analysis. Electropherogram of (**A**) mPCR amplicons and (**B**) fluorescent curves of mqPCR. Nt—no template control; x-axis—number of PCR cycles; y-axis—delta Rn (fluorescence).

PCRs for identification of beef, pork, horse and poultry (chicken, turkey) meat were designed and experimentally verified in our previous work; used PCRs were selective and allowed the detection of 30 copies of the haploid pig genome, 26 copies of the haploid beef-cattle genome and 11 copies of haploid chicken genome [24]. The limit of quantification for the mqPCR system was 12.5 ng of DNA per reaction. Four types of meat products were analyzed in this work: ham, Czech sausage, meat in its natural juices and luncheon meat. The results of mPCR and mqPCR analysis are shown in Table 4, examples of primary results in Figure 6.

Analyses of hams by quadruplex PCR of mtDNA and multiplex qPCR of genomic DNA gave the same results, the presence of the declared animal species (i.e., chicken or pig) was established. Eleven samples of typical Czech sausages were analyzed by mPCR, with identical results obtained for both target DNA (mtDNA and gDNA). The presence of the animal species declared in the label (i.e., beef and/or pork) was proved. Six cans of meat in natural juices were analyzed, five were made mainly from pork and one was made mainly from beef. Results that matched the labels were obtained for both target DNAs (mtDNA and gDNA) for all five pork cans. Beef meat in natural juices (sample 27) also contained mechanically separated poultry meat and pork skin, all three animal´s DNA was detected by mqPCR with gDNA as a target. It worked despite the heat treatment. Seven different Luncheon meats were analyzed, all the results obtained by mqPCR with gDNA as a target corresponded to the declaration on the label. A discrepancy between the results of mtDNA and gDNA analysis was observed in this type of meat product (luncheon meat). White adipose tissue (univacuolar adipocytes) contains a low number of mitochondria. The use of mqPCR amplified gDNA, which enables the better detection of a low amount of target DNA, is recommended for the analysis of samples containing large quantities of fat (e.g., luncheon meat).

Results obtained by PCR analyses of mtDNA and gDNA were the same for ham and sausages; for highly processed samples (cans) only PCRs targeting the gDNA appeared to be appropriate. Four samples of luncheon meat (21, 23, 24 and 26) and one meat in natural juices (27) showed differences in result of mtDNA and gDNA analyses, these samples were also categorized as a “suspicious sample” by DART–HRMS as explained in Section 3.1.2. The DART–HRMS analysis is a quick, cheap and high-throughput compare to moderate time-consuming PCRs (See Table 5). Therefore, DART–HRMS was proposed to be used as a screening method followed by through, a highly accurate, more laborious and more expensive amplification of gDNA by mqPCR only of suspicious samples. The advantages of such combinations are time, money and laboratory personnel-capacity saving.

The results of mqPCR analyses were identical to the declarations by the manufacturer for the product labels. This fact is quite encouraging, considering the relatively common occurrence of fraudulent food practices, including for meat products. One possible explanation is that we focused on analyzing regional sausages and luncheon meats. The traditional (original) recipe for sausage and luncheon meat does not allow the addition of chicken meat (it is forbidden) and it can be said that the manufacturers did not follow these rules when preparing these regional sausages. With luncheon meat, almost half of the products analyzed were mislabeled.

The requirements are set out in Act No. 326/2001 Coll., issued by the Czech Ministry of Agriculture, which imposes requirements for meat labeling. Ham is made from the musculature of pork, poultry or beef, a heat-treated product. As for sausages, in this study, typical Czech sausages called “Špekáčky” were analyzed. These are a kind of sausage made from a finely minced mixture of pork and beef with smoked bacon pieces that gave the product its name. The true sausage according to the original recipe should consist of 50% beef, 20% pork cut out of the skin and 30% chopped bacon pieces. The basic raw materials according to current regulations are beef, pork or veal; a minimum of 40% meat and a maximum of 45% fat are required, and it does not allow the use of mechanically separated meat and poultry. The last two products were canned, i.e., products hermetically sealed in packaging and sterilized. According to the current regulation, canned food called “pork in natural juices” must contain at least 70% meat. The water content must not exceed 70% and the fat may be up to 40%. Canned food called “beef in natural juices” must contain at least 70% meat, the water content must not exceed 80%, and the fat may be up to 20%. The sum of all parameters exceeds 100% due to the fact that much of the fat and water in the product comes from the meat used. Luncheon meat should be made from pork and beef. The law specifies pork luncheon meat, which has the same limits on meat, fat and water content as “pork in natural juices”.

#### 3.1.5. Comparison of Methods

Commonly used molecular genetic methods such as PCR are based on the isolation and analysis of different DNA types, while the new innovative DART–HRMS analytical method is focused on the analysis of TAGs profiles. PCRs use DNA markers, which are unique, stable, well-known and independent of the type of animal breeding. Detection/quantification of these DNA markers can be used also for the investigation of mixed animal meat products. The quality and quantity of isolated DNA is the crucial parameter for the successful use of molecular genetic methods. There are many different procedures for isolating and analyzing DNA, which differ in the yield, cost and time requirements of the procedure. In PCR analysis, the whole procedure usually takes hours. Sample preparation for DART–HRMS is very simple and fast; the DART–HRMS analysis itself requires only a few seconds. On the other hand, the evaluation of the obtained data are significantly simplified with PCR analysis compared to the use of advanced chemometric analysis in the DART–HRMS evaluation procedure. With the DART–HRMS process, up to 130 samples per hour can be measured, while these 130 samples can take at least one working day to be analyzed by mPCR. On the other hand, PCR provides accurate results and is considered an arbitrary method, whereas the DART–HRMS method is primarily a screening method for testing a large number of samples and selecting only suspect specimens, which are then confirmed by DNA analysis (Table 5).

## 4. Conclusions

In the presented work the multiplex mPCR analysis of DNA and DART–HRMS analysis of the TAGs profile of meat (pork, beef, chicken) in combination with multivariate statistical analysis were compared theoretically and practically. In both cases, comparable results were obtained. DART–HRMS is a quick method with a great potential for screening a large number of samples. PCRs analysis can be used for precise animal species identification; however, it takes more time to get results.

Our results suggest that DART–HRMS could be used primarily as a screening method and suspected samples could be subsequently analyzed by PCR method. This combination of both approaches has potential for meat type verification or detection of adulteration, respectively.

## Figures and Tables

**Table 1:** Declared composition of analyzed samples.

Sample	Product	Pork Meat/Lard (%)	Chicken Meat (%)	Beef Meat (%)	Max Fat Content (%)
1	Chicken ham	–	92	–	1.5
2	Poultry ham	–	60	–	1.5
3	Pork ham	95	–	–	10
4	Sausage	16/Y	–	35	40
5	Sausage	40/Y	–	10	40
6	Sausage	62/Y	–	23	40
7	Sausage	40/25	–	10	34
8	Sausage	54/Y	–	26	44
9	Luncheon meat	N/Y	–	Y	40
10	Sausage	16/Y	–	35	45
11	Sausage	17/Y	–	26	N
12	Sausage	17.5/Y	–	38.5	45
13	Meat in natural juices	70	–	–	33
14	Luncheon meat	79	–	–	30
15	Meat in natural juices	92	–	–	N
16	Meat in natural juices	70	–	–	40
17	Meat in natural juices	70	–	–	30
18	Sausage	33/30	–	22	N
19	Sausage	71/Y	–	16	45
20	Sausage	43/30	–	17	45
21	Luncheon meat	48	Y	–	40
22	Meat in natural juices	30 + MSM/Y		–	N
23	Luncheon meat	18	32	–	30
24	Luncheon meat	35	30	–	25
25	Luncheon meat	71		–	40
26	Luncheon meat	31/Y	39	–	26
27	Meat in natural juices	Y	Y	70	27

Y—label on packaging indicates the usage, but the percent content is not stated; N—not labeled on the packaging; MSM—mechanically separated meat.

**Table 2:** Primers and probes used in this study.

Meat Species	Name of Primer	Target Sequence	Sequence of Primer [5′-3′]	Product [bp]	References
Universal F	SIM	Cytochrome b (mtDNA)	GACCTCCCAGCTCCATCAAACATCTCATCTTGATGAAA		[12]
Beef R	B		CTAGAAAAGTGTAAGACCCGTAATATAAG	274	[12]
Pork R	P		GCTGATAGTAGATTTGTGATGACCGTA	398	[12]
Chicken, turkey R	C		CGTATTGTACGTTCCGGCAAG	169	[24]
Horse R	H		CTCAGATTCACTCGACGAGGGTAGTA	439	[12]
Beef	Bos-PDE-f	Cyclic-GMP-phospho-diesterase (gDNA)	ACTCCTACCCATCATGCAGAT	104	[11,25]
	Bos-PDE-r		TGTTTTTAAATATTTCAGCTAAGAAAAA		
	Bos-PDE-p		TexasRed: AACATCAGGATTTTTGCTGCATTTGC:BHQ2		
Pork	Sus1-F	Beta-actin (gDNA)	CGAGAGGCTGCCGTAAAGG	107	[11,26]
	Sus1-R		TGCAAGGAACACGGCTAAGTG		
	Sus1-p		HEX:TCTGACGTGACTCCCCGACCTGG:BHQ1		
Mammals and poultry	MY-F	Myostatin (gDNA)	TTGTGCAAATCCTGAGACTCAT	97	[11,27]
	MY-R		ATACCAGTGCCTGGGTTCAT		
	My-p		FAM:CCCATGAAAGACGGTACAAGGTATACTG:BHQ1		
Chicken	ChIn-F	Interleukin-2 (gDNA)	TGTTACCTGGGAGAAGTGGTTACT	135	[25]
	ChIn-R		CTGACCATAAAGAATACCTACCG		[24]
	ChIn-p		TAMRA:TGAAGAAAGAAACTGAAGATGACACTGAAATTAAAG:BHQ2		[25]

**Table 3:** Characteristic ions for pork, beef and chicken.

*m*/*z*	Δppm	Formula	Identification	Significant Ions for
846.7535	1.259	C_53_H_100_NO_6_	C 50:3	chicken
848.7682	1.339	C_53_H_102_NO_6_	C 50:2	chicken/pork
850.7836	1.559	C_53_H_104_NO_6_	C 50:1	chicken/pork
852.7975	4.820	C_53_H_106_NO_6_	C 50:0	chicken/pork
872.7683	2.104	C_55_H_102_NO_6_	C 52:4	chicken
874.7837	1.562	C_55_H_104_NO_6_	C 52:3	chicken
876.7995	1.672	C_55_H_106_NO_6_	C 52:2	pork/beef
878.8130	2.522	C_55_H_108_NO_6_	C 52:1	pork/beef
880.8246	4.112	C_55_H_110_NO_6_	C 52:0	pork/beef
896.7689	1.412	C_57_H_102_NO_6_	C 54:6	chicken
898.7841	1.898	C_57_H_104_NO_6_	C 54:5	chicken
900.7996	2.050	C_57_H_106_NO_6_	C 54:4	chicken
902.8148	3.636	C_57_H_108_NO_6_	C 54:3	pork/beef
904.8306	1.709	C_57_H_110_NO_6_	C 54:2	beef
906.8457	2.974	C_57_H_112_NO_6_	C 54:1	beef

**Table 4:** Results of meat sample by DNA analysis.

	Product	Declared Composition			mPCR			mqPCR		
		Pork	Chicken	Beef	Pork	Chicken/Turkey	Beef	Pork	Chicken	Beef
1	Chicken ham	**-**	**+**	**-**	**-**	**+**	**-**	**-**	**+**	**-**
2	Poultry ham	**-**	**+**	**-**	**-**	**+**	**-**	**-**	**+**	**-**
3	Pork ham	**+**	**-**	**-**	**+**	**-**	**-**	**+**	**-**	**-**
4	Sausage	**+**	**-**	**+**	**+**	**-**	**+**	**+**	**-**	**+**
5	Sausage	**+**	**-**	**+**	**+**	**-**	**+**	**+**	**-**	**+**
6	Sausage	**+**	**-**	**+**	**+**	**-**	**+**	**+**	**-**	**+**
7	Sausage	**+**	**-**	**+**	**+**	**-**	**+**	**+**	**-**	**+**
8	Sausage	**+**	**-**	**+**	**+**	**-**	**+**	**+**	**-**	**+**
9	Luncheon meat	**+**	**-**	**+**	**+**	**-**	**+**	**+**	**-**	**+**
10	Sausage	**+**	**-**	**+**	**+**	**-**	**+**	**+**	**-**	**+**
11	Sausage	**+**	**-**	**+**	**+**	**-**	**+**	**+**	**-**	**+**
12	Sausage	**+**	**-**	**+**	**+**	**-**	**+**	**+**	**-**	**+**
13	Meat in natural juices	**+**	**-**	**-**	**+**	**-**	**-**	**+**	**-**	**-**
14	Luncheon meat	**+**	**-**	**-**	**+**	**-**	**-**	**+**	**-**	**-**
15	Meat in natural juices	**+**	**-**	**-**	**+**	**-**	**-**	**+**	**-**	**-**
16	Meat in natural juices	**+**	**-**	**-**	**+**	**-**	**-**	**+**	**-**	**-**
17	Meat in natural juices	**+**	**-**	**-**	**+**	**-**	**-**	**+**	**-**	**-**
18	Sausage	**+**	**-**	**+**	**+**	**-**	**+**	**+**	**-**	**+**
19	Sausage	**+**	**-**	**+**	**+**	**-**	**+**	**+**	**-**	**+**
20	Sausage	**+**	**-**	**+**	**+**	**-**	**+**	**+**	**-**	**+**
21	Luncheon meat	**+**	**+**	**-**	**-** ^ᵻ^	**+**	**-**	**+**	**+**	**-**
22	Meat in natural juices	**+**	**-**	**-**	**+**	**-**	**-**	**+**	**-**	**-**
23	Luncheon meat	**+**	**+**	**-**	**+**	**+**	**+** ^ᵻ^	**+**	**+**	**-**
24	Luncheon meat	**+**	**+**	**-**	**-** ^ᵻ^	**+**	**-**	**+**	**+**	**-**
25	Luncheon meat	**+**	**-**	**-**	**+**	**-**	**-**	**+**	**-**	**-**
26	Luncheon meat	**+**	**+**	**-**	**+**	**+**	**+** ^ᵻ^	**+**	**+**	**-**
27	Meat in natural juices	**+**	**+**	**+**	**+**	**+**	**-** ^ᵻ^	**+**	**+**	**+**

Legend: **+** amplicon present; **-** amplicon not detected; **^ᵻ^** - difference in results of mPCR and mqPCR analysis.

**Table 5:** Comparison of DART–HRMS and PCR.

Parameters	DART–HRMS	PCR
Target molecule	Triacylglycerols	DNA
Preparation step	Hexane extract–lipophilic fraction containing triacylglycerols	DNA isolation—many methods available
Capacity of the machine	High-throughput method (+++)	Mainly 96 reactions in one run (++)
Cost of the analysis (only the retail price of chemicals is included)	Very low (+++)	Low (++)
Duration	Extraction: moderate (+++) Analysis: quick (+++) Evaluation: long	Extraction: moderate (++) Analysis: moderate (++) Evaluation: quick (+++)
Price of the required instrumentation	High (-)	Low for classical PCR instrument, moderate for qPCR device (+)
Feasibility for analysis of raw products	Yes (+++)	Yes, reliable (+++)
Feasibility for analysis of heat-treated meat products	Yes (++)	Yes, reliable (+++)
Feasibility for analysis of mixtures	Yes (++)	Yes, reliable (+++)
Feasibility for analysis of products containing high amounts of fat	Yes (+++)	Yes, gDNA is recommended as a target (+++)
Conduction of the experiment	Laboratory device (-), performing the analysis (+++)	Laboratory device (++), performing the analysis (+)
Claims for evaluation of results	Demanding for evaluation–experience is needed, because of the statistical analysis.	Simple to evaluate (+++). The PCR amplicon or fluorescence curve is or is not there, which is clearly visible from the primary results
Usage	Screening method	Confirmatory method

## References

[1] Hsieh Y.P., Woodward B.B., Ho S.H. (1995). Detection of Species Substitution in Raw and Cooked Meats Using Immunoassays. J. Food Prot..

[2] Ballin N.Z., Vogensen F.K., Karlsson A.H. (2009). Species determination—Can we detect and quantify meat adulteration?. Meat Sci..

[3] Fajardo V., González I., Rojas M., García T., Martín R. (2010). A review of current PCR-based methodologies for the authentication of meats from game animal species. Trends Food Sci. Technol..

[4] Köppel R., Daniels M., Felderer N., Brünen-Nieweler C. (2013). Multiplex real-time PCR for the detection and quantification of DNA from duck, goose, chicken, turkey and pork. Eur. Food Res. Technol..

[5] Iwobi A., Sebah D., Spielmann G., Maggipinto M., Schrempp M., Kraemer I., Gerdes L., Busch U., Huber I. (2017). A multiplex real-time PCR method for the quantitative determination of equine (horse) fractions in meat products. Food Control.

[6] Griffiths A.M., Sotelo C.G., Mendes R., Pérez-Martín R.I., Schröder U., Shorten M., Silva H.A., Verrez-Bagnis V., Mariani S. (2014). Current methods for seafood authenticity testing in Europe: Is there a need for harmonisation?. Food Control.

[7] Murugaiah C., Al-Talib H., Radu S. (2015). Forensics: Food Authentication Using MtDNA. J. Nutr. Health Food Sci..

[8] Mozola M.A., Peng X., Wendorf M., Artiga L. (2007). Evaluation of the GeneQuence^®^ DNA hybridization method in conjunction with 24-h enrichment protocols for detection of Salmonella spp. in select foods: Collaborative study. J. AOAC Int..

[9] Tichoniuk M., Ligaj M., Filipiak M. (2008). Application of DNA hybridization biosensor as a screening method for the detection of genetically modified food components. Sensors.

[10] Chapela M.J., Sotelo C.G., Calo-Mata P., Pérez-Martín R.I., Rehbein H., Hold G.L., Quinteiro J., Rey-Méndez M., Rosa C., Santos A.T. (2002). Identification of Cephalopod Species (Ommastrephidae and Loliginidae) in Seafood Products by Forensically Informative Nucleotide Sequencing (FINS). J. Food Sci..

[11] Iwobi A., Sebah D., Kraemer I., Losher C., Fischer G., Busch U., Huber I. (2015). A multiplex real-time PCR method for the quantification of beef and pork fractions in minced meat. Food Chem..

[12] Matsunaga T., Chikuni K., Tanabe R., Muroya S., Shibata K., Yamada J., Shinmura Y. (1999). A quick and simple method for the identification of meat species and meat products by PCR assay. Meat Sci..

[13] Barbuto M., Galimberti A., Ferri E., Labra M., Malandra R., Galli P., Casiraghi M. (2010). DNA barcoding reveals fraudulent substitutions in shark seafood products: The Italian case of “palombo” (*Mustelus* spp.). Food Res. Int..

[14] Thakur M., Singh S., Shukla M., Sharma L., Agarwal N., Goyal S., Sambandam S. (2012). Identification of Galliformes through Forensically Informative Nucleotide Sequencing (FINS) and its Implication in Wildlife Forensics. J. Forensic Res..

[15] Jírů M., Stranska M., Kocourek V., Krmela A., Tomaniová M., Rosmus J., Hajslova J. (2019). Authentication of meat species and net muscle proteins: Updating of an old concept. Czech J. Food Sci..

[16] Ruiz Orduna A., Husby E., Yang C.T., Ghosh D., Beaudry F. (2015). Assessment of meat authenticity using bioinformatics, targeted peptide biomarkers and high-resolution mass spectrometry. Food Addit. Contam. Part A.

[17] Cody R.B., Laramée J.A., Durst H.D. (2005). Versatile new ion source for the analysis of materials in open air under ambient conditions. Anal. Chem..

[18] Hajslova J., Cajka T., Vaclavik L. (2011). Challenging applications offered by direct analysis in real time (DART) in food-quality and safety analysis. TrAC Trends Anal. Chem..

[19] Worley B., Powers R. (2013). Multivariate Analysis in Metabolomics. Curr. Metab..

[20] Blasco H., Błaszczyński J., Billaut J.C., Nadal-Desbarats L., Pradat P.F., Devos D., Moreau C., Andres C.R., Emond P., Corcia P. (2015). Comparative analysis of targeted metabolomics: Dominance-based rough set approach versus orthogonal partial least square-discriminant analysis. J. Biomed. Inform..

[21] Berrueta L.A., Alonso-Salces R.M., Héberger K. (2007). Supervised pattern recognition in food analysis. J. Chromatogr. A.

[22] Triba M.N., Le Moyec L., Amathieu R., Goossens C., Bouchemal N., Nahon P., Rutledge D.N., Savarin P. (2015). PLS/OPLS models in metabolomics: The impact of permutation of dataset rows on the K-fold cross-validation quality parameters. Mol. Biosyst..

[23] ISO (2005). Foodstuffs-Methods of Analysis for the Detection of Genetically Modified Organisms and Derived Products-Nucleic Acid Extraction.

[24] Zdeňková K., Akhatova D., Fialová E., Krupa O., Kubica L., Lencová S., Demnerová K. (2018). Detection of meat adulteration: Use of efficient and routine-suited multiplex polymerase chain reaction-based methods for species authentication and quantification in meat products. J. Food Nutr. Res..

[25] Laube I., Zagon J., Spiegelberg A., Butschke A., Kroh L.W., Broll H. (2007). Development and design of a ‘ready-to-use’ reaction plate for a PCR-based simultaneous detection of animal species used in foods. Int. J. Food Sci. Technol..

[26] Köppel R., Ruf J., Zimmerli F., Breitenmoser A. (2008). Multiplex real-time PCR for the detection and quantification of DNA from beef, pork, chicken and turkey. Eur. Food Res. Technol..

[27] Laube I., Spiegelberg A., Butschke A., Zagon J., Schauzu M., Kroh L., Broll H. (2003). Methods for the detection of beef and pork in foods using real-time polymerase chain reaction. Int. J. Food Sci. Technol..

[28] Hurkova K., Uttl L., Rubert J., Navratilova K., Kocourek V., Stranska-Zachariasova M., Paprstein F., Hajslova J. (2019). Cranberries versus lingonberries: A challenging authentication of similar Vaccinium fruit. Food Chem..

[29] Hrbek V., Rektorisova M., Chmelarova H., Ovesna J., Hajslova J. (2018). Authenticity assessment of garlic using a metabolomic approach based on high resolution mass spectrometry. J. Food Compos. Anal..

